# A PowerPack of SuperScientists: An innovative concept by African scientists to address gender bias and inequity in science

**DOI:** 10.12688/wellcomeopenres.17668.2

**Published:** 2023-02-24

**Authors:** Maphe Mthembu, Omolara Baiyegunhi, Yanga Mdleleni, Lerato Ndlovu, Hannah Keal, Kim Waddilove, Justin C. Yarrow, Victoria Kasprowicz, Thumbi Ndung'u, Emily B. Wong

**Affiliations:** 1 Africa Health Research Institute, Durban, 4001, South Africa; 2 University of KwaZulu-Natal, Nelson R. Mandela School of Medicine, Durban, 4001, South Africa; 3 HIV Pathogenesis Programme, The Doris Duke Medical Research Institute, University of KwaZulu-Natal, Durban, 4001, South Africa; 4 CodeMakers NPC - SuperScientists, Durban, 4001, South Africa; 5 Ragon Institute of MGH, MIT and Harvard, Cambridge, MA, 02139-3583, USA; 6 Harvard Medical School, Boston, MA, 02115, USA; 7 Division of Infection and Immunity, University College London, London, WC1E 6BT, UK; 8 Division of infectious Diseases, Heersink School of Medicine, University of Alabama at Birmingham, AL, 35294, USA

**Keywords:** Gender equity, African science, Intersectionality, Bias, Early career scientists

## Abstract

Underrepresentation of women in scientific leadership is a global problem. To understand and counter narratives that limit gender equity in African science, we conducted a public engagement campaign. Scientists representing six sub-Saharan African countries and multiple career stages used superhero imagery to create a diverse and unified team advocating for gender equity in science. In contrast to many traditional scientific environments and global campaigns, this “PowerPack of SuperScientists” was led by early-career Black female scientists whose perspectives are often under-represented in discussions about gender equity in science. The superhero imagery served as a powerful and fun antidote to imposter syndrome and helped to subvert traditional power structures based on age, race and sex. In an interactive social media campaign, the PowerPack developed insights into three themes: a) cultural stereotypes that limit women’s scientific careers, b) the perception of a “conflict” between family and career responsibilities for women scientists, and c) solutions that can be adopted by key stakeholders to promote gender equity in African science. The PowerPack proposed solutions that could be undertaken by women working individually or collectively and interventions that require allyship from men, commitment from scientific institutions, and wider societal change. Further work is required to fully engage African scientists from even more diverse and disadvantaged backgrounds and institutions in these solutions and to enhance commitment by different stakeholders to achieving gender equity in science. Our experience suggests that creative tools should be used to subvert power dynamics and bring fresh perspectives and urgency to this topic.

## Disclaimer

The views expressed in this article are those of the author(s). Publication in Wellcome Open Research does not imply endorsement by Wellcome.

## Background

Throughout the world, and in Africa, women are under-represented in science ^1^. The well-worn analogy of the “leaky pipeline” illustrates that a great number of women scientists are “lost” to science at various points along their career, resulting in fewer women leading scientific publications, being awarded prestigious grants, faculty, and leadership positions ^2^.

Historical and systemic factors prevent women from pursuing and thriving in scientific careers ^3– 5^. Women occupy over 70% of global health positions but less than 25% of leadership positions ^6^. Even in countries with highly diverse populations, gender and racial disparities interact to decrease diversity in the scientific workforce. In the USA, scientific faculty and leadership positions are dominated by men and white people while Black and Hispanic women are poorly represented in these ranks ^7^. The sub-Saharan Africa (SSA) Science Granting Council (SGC) who are key players of African science innovation were also reported to have more man than women holding positions across most levels of employment especially leadership positions ^8^. The underrepresentation of women, and in particular Black women, in scientific leadership positions costs science and society. Limited representation of women in scientific leadership hampers the ambitions of girls seeking career role models and the practical advice and help available to women seeking to advance their scientific careers. The insights, voices and perspectives of women from diverse backgrounds are under-represented among circles of scientific influence. The overall quality of science is impacted because innovative ideas stem from teams that represent diverse perspectives ^9^. The achievement of gender diversity in science has been linked to substantial increases in scientific outputs, creativity and innovation ^6, 9^.

The coronavirus disease 2019 (COVID-19) pandemic has further exacerbated existing gender inequalities in science ^10^. During the pandemic, women have experienced unique pressures and childcare demands on their time resulting in decreased scientific authorship by women ^11, 12^. There is an urgent need for radical solutions to promote diversity in science, especially in scientific leadership.

We describe a project that was established to spark dialogue among scientists whose voices are often side-lined due to intersecting race and gender power dynamics. Our aim was to reframe narratives that contribute to gender inequity in science, by re-imagining African scientists at all career stages as superheroes working as a team to advocate for the idea of gender equity in science. Here, we provide details about our online campaign and its insights into potential solutions for achieving gender equity in African science.

## Approach

**Figure 1.**
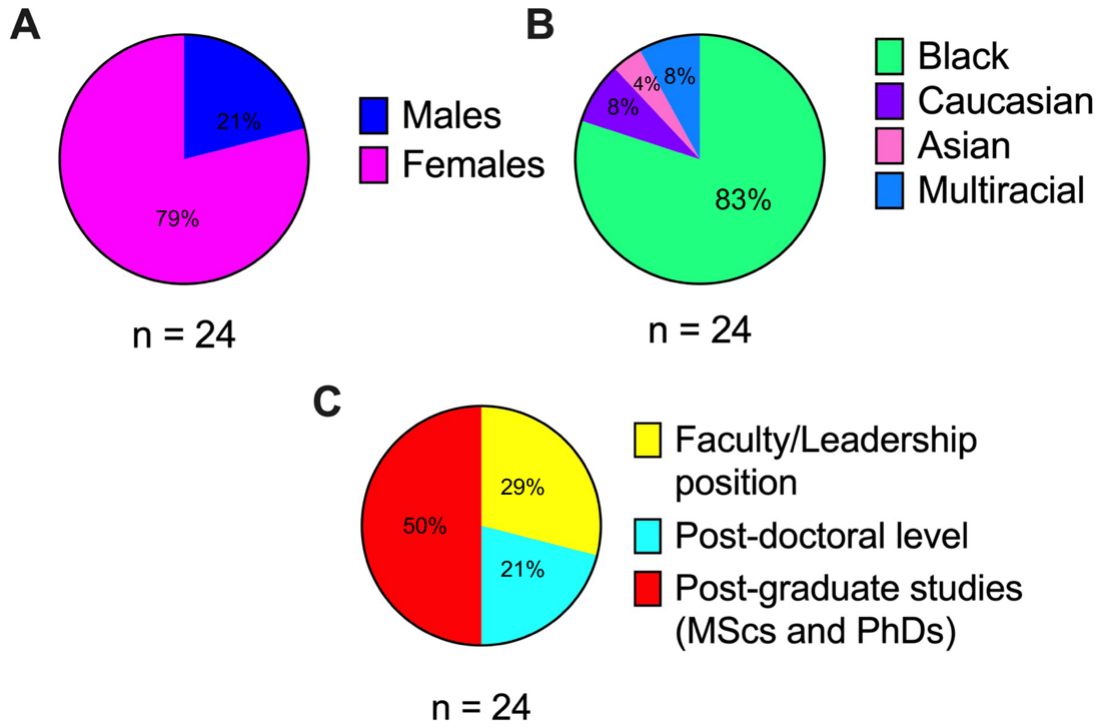
Characteristics of a group of scientists spearheading the gender equity public engagement campaign. ( **A**) Gender distribution of this group; females (pink) and males (blue). ( **B**) Racial distribution of this group; Black (green), Caucasian (purple), Asian (pink), Multiracial (blue). ( **C**) Career-stage distribution of this group; faculty/leadership (yellow), postdoctoral trainee (turquoise), post-graduate student (red).

**Figure 2.**
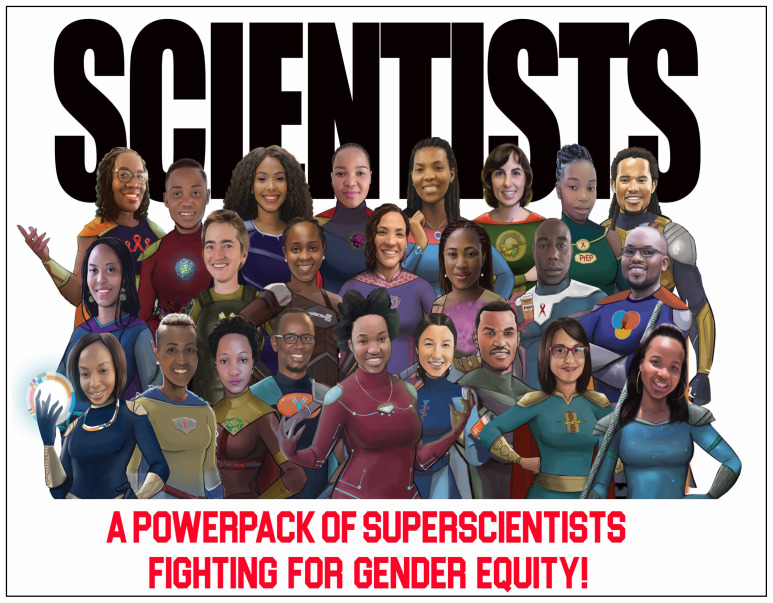
The PowerPack of SuperScientists in the fight for gender equity in science.

We engaged in a collaborative project involving the Sub-Saharan African Network for TB and HIV Excellence (SANTHE) and Codemakers, a South African science education non-profit organization. Scientists from across the SANTHE network were invited to participate in the project and using intersectionality lens, we selected a group that represented diverse career-stages, gender and nationality (in the Sub-Saharan African region) while intentionally overrepresenting historically disempowered groups, like early-career Black female scientists. The selected group consisted of 24 scientists representing 6 Sub-Saharan African countries (South Africa, Kenya, Rwanda, Uganda, Botswana, and Zambia). Scientists were 79% female and 21% male ( Figure 1A) and of diverse racial origin, with 80% Black, 8% Caucasian, 4% Asian and 8% multi-racial ( Figure 1B). They represented multiple career levels, with 29% in faculty or leadership positions, 21% at the post-doctoral level, and 50% post-graduate students (master’s and PhDs) ( Figure 1C). Becoming a SuperScientist involved each scientist reflecting on their scientific "brand", identifying their strengths as scientists (‘superpowers’), and designing a superhero persona complete with costumes and insignias. After being transformed into superheroes, the group of scientists were referred to as the ‘PowerPack of SuperScientists’ ( Figure 2).

**Figure 3.**
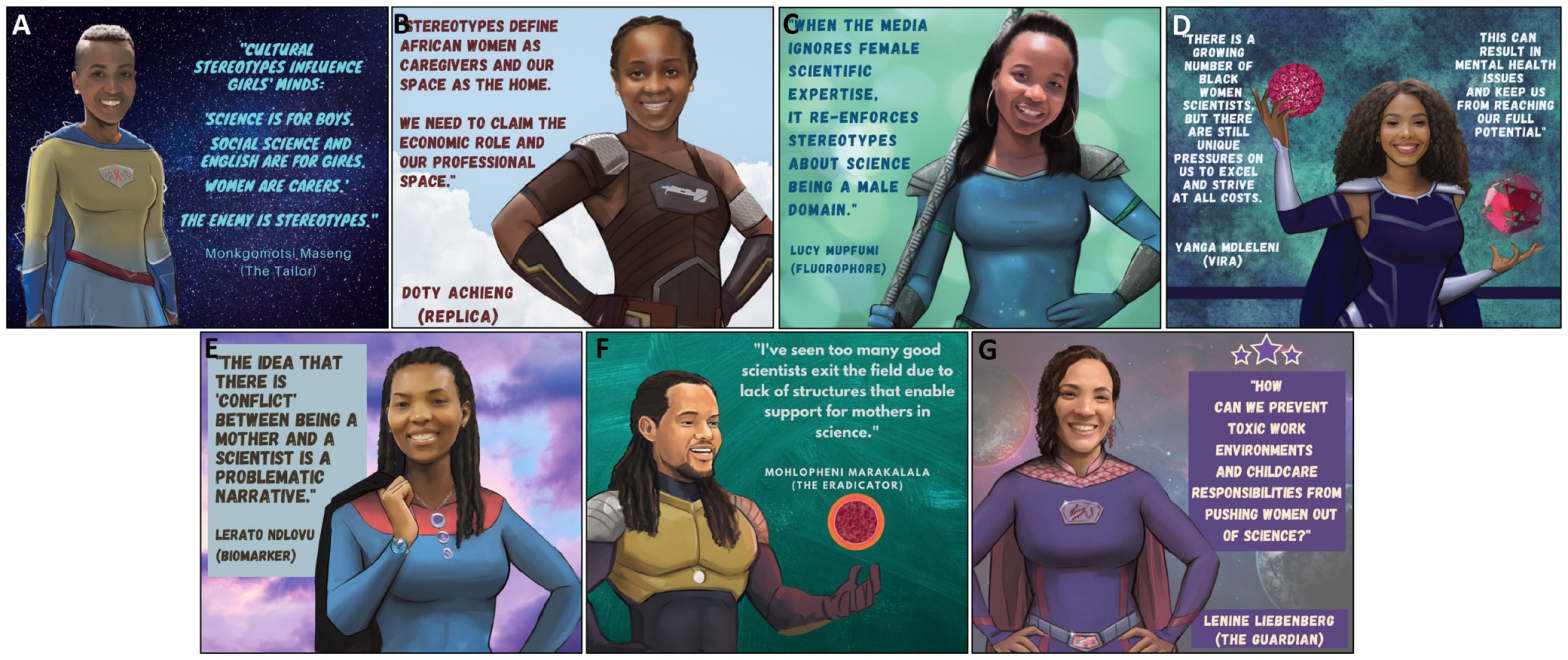
Cultural stereotypes and problematic narratives that uphold gender inequity in science.

During a series of closed sessions, the PowerPack discussed their experiences with gendered narratives in science and co-created the themes of the campaign, deciding to focus on specific barriers and suggested solutions to achieving gender equity in science. The team then conducted a month-long social media and online campaign, “African Scientists for Gender Equity,” on Facebook (@GenderEquitySci), Twitter (@genderequitysci) and Zoom that introduced PowerPack members on social media using their SuperScientist imagery and quotations that encapsulated the campaign themes ( Figure 3– Figure 6). The campaign also included two interactive webinars. Surviving and Thriving in Science in 2020 targeted an audience of young African women in science and featured three female scientists who have negotiated barriers to gender equity to achieve successful careers. Solutions for Gender Equity targeted an audience across the African scientific community and featured 3 thought-leaders from the continent who discussed how gender equity in science could be promoted at the institutional level. The month-long campaign yielded over 1,000 followers and 250,000 impressions from across the world.

**Figure 4.**
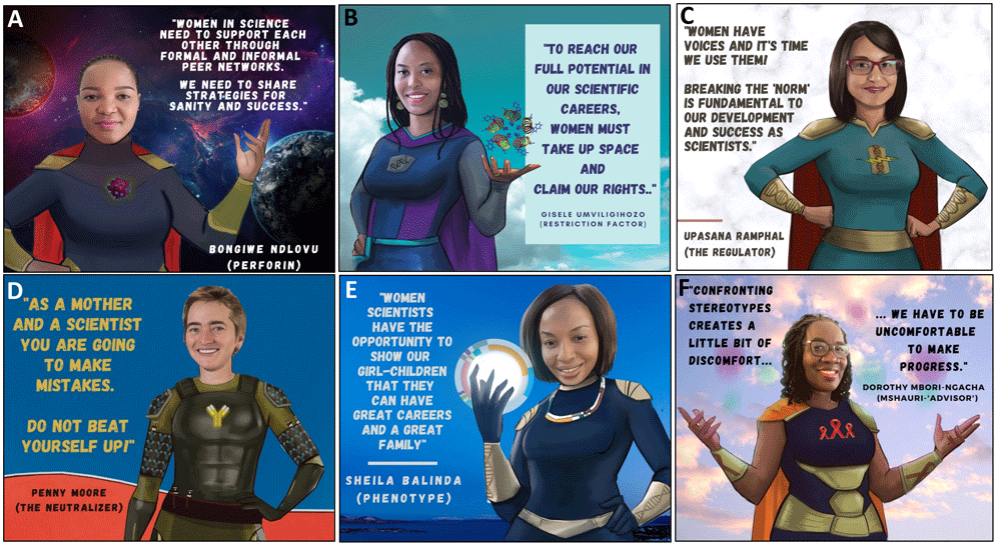
Tools that women can use to promote gender equity in science.

**Figure 5.**
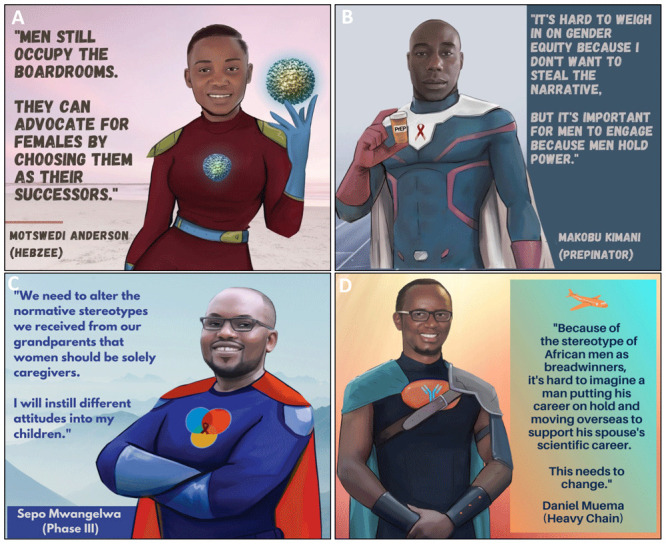
Tools that men can use to promote gender equity in science.

**Figure 6.**
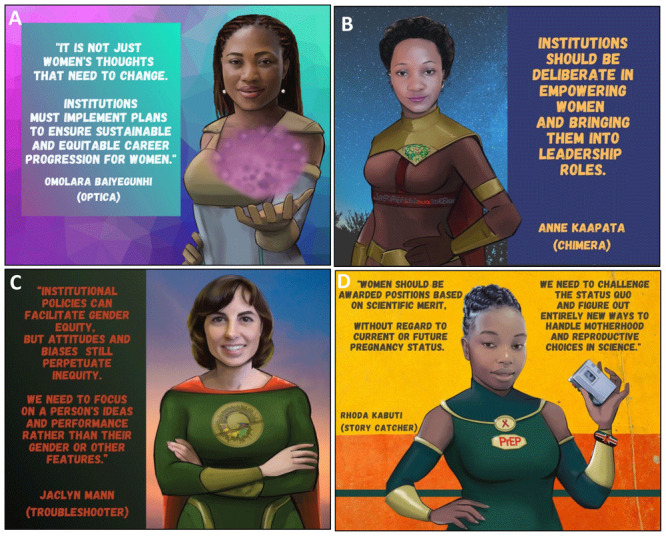
The role of scientific institutions in promoting gender equity in science.

## African scientists for gender equity campaign themes

The campaign themes are summarized in Table 1. Discussions highlighted gender equity barriers and suggested solutions that could be applied at individual, environmental and institutional levels.

**Table 1.** Barriers to gender equity in science and suggested solutions.

Levels	Identified barriers	Suggested solutions
**Individual**	Internalized gendered cultural stereotypes Isolation within the scientific environment. Pressure to achieve scientific excellence and its impact on mental health Self-doubt and self-judgement about the ability to negotiate the “conflict” between motherhood and science	Learn to recognize and actively oppose negative scripts and judgements Female scientists must find their voice, take up space and seek peer support and mentorship. Recognize the impact of intersecting racial and gender identities on stress and seek mental health care. Mother scientists must expect imperfection in motherhood and their scientific career and accept help in both roles
**Environmental /** **Societal**	Limited representation of successful female scientists Societal expectations that women bear responsibility for the domestic sphere Patriarchy and post-colonial dynamics in the society	Science and the society should celebrate scientists of both genders and scientist-mothers Male scientists and the male partners of female scientists should embrace hands-on parenting, including paternity leave. Acknowledge these and their multiple impacts on gender inequity in science Scientific society and fellow women should promote women leaders
**Institutional**	Lack of support for the specific needs of scientist-mothers, especially those in vulnerable/early stages of their scientific careers Continued attrition of women scientists at all career levels (“leaky pipeline”) Gender equity in science perceived as the exclusive responsibility of women	Clear and publicized maternity leave policies for all career levels. Supplemental funds for continued productivity during maternity leave, extra childcare during travel. Clean and comfortable lactation facilities. Enact policies to promote equal pay, positions and funding for both genders. Track institutional performance outcomes. Promote women scientists to leadership. Men should embrace gender equity and participate in campaigns.

### Barriers to gender equity in Sub-Saharan Africa science

Barriers to gender equity in science have been reviewed extensively and a comprehensive discussion is beyond the scope of this report ^5, 13, 14^. However, in reflecting on gender dynamics in African science, the SuperScientists drew on lived experiences and highlighted specific barriers relevant to the scientific context of Sub-Saharan Africa. These barriers can have negative impacts on women’s careers at multiple levels: individual, environmental and institutional.

***a. Cultural stereotypes that limit women’s scientific careers*.** Culture encompasses customs, beliefs, attitudes, behaviors and traditions ^15^. Cultures are specific to societies, and they manifest and influence how we understand ourselves and navigate the world around us ^15^. Our SuperScientists came from diverse backgrounds and different countries, and therefore experienced different cultural norms in their upbringing and their current circumstances. Even though we had a diverse group, it is important to highlight that due to the scope of the current project we could only focus on the sub-Saharan African countries, excluding the North and West Africa. Due to their inclusion in the SANTHE network, the selected group of participants may be more privileged and better-funded than others from African scientific institutions outside of the network. We therefore acknowledge the gaps in our findings and may have missed important insights from unrepresented and less advantaged scientific institutions from across the continent. Nonetheless, from our group, many PowerPack members highlighted the multifaceted marginalization of African women that prohibits them from pursuing and thriving in science careers. Like many other cultures, most African societies are patriarchal; privileging and empowering boys and men over girls and women ^16, 17^. A recurring theme from the PowerPack was that cultural stereotypes contribute to gender inequity in science. PowerPack members reported that in many of their cultures of origin, women were traditionally associated with domestic rather than professional environments, resulting in scant experience or imagery to associate women with scientific careers ( Figure 3A–B). PowerPack members reported being raised in cultures that subordinated young girls and women to boys and men and that these experiences caused stereotypes to become entrenched in their minds from a young age. For instance, in the context of the current COVID-19 pandemic, media coverage of mostly male scientists muted female scientists’ perspectives on the pandemic and furthered societal stereotypes ( Figure 3C) ^18^.

Although the identified stereotypes have their origin in patriarchal and colonial society, many members of the campaign acknowledged some level of internalization of these biases. Language and geographical location influence cultural behaviors and continue to dictate where we find ourselves in science and these colonial legacies continue to limit ambitions and opportunities especially for women. Women scientists from the PowerPack reported associating these internalized gender stereotypes from their upbringing with current struggles to express their true feelings and discomforts in male dominated spaces. These dynamics limited their ability to address social ills and claim space in male dominated situations, including their scientific environments ^19^. Thus, members of the PowerPack reported that cultural dynamics resulted in internalized stereotypes and barriers to the pursuit of gender equity in science. Upon reflection, PowerPack members recognized that gendered cultural expectations have become unconscious frames that shape their behaviors, plans and aspirations. Recent SSA based studies also highlighted socio-cultural behaviors as one of the major drivers of gender inequitable scientific career progression ^20, 21^. Moreover, some of these stereotypes have power to create gender inequity in science because they are internalized and unconscious. The fact that they are often unrealized and unspoken, especially in scientific environments, makes it hard for them to be acknowledged and addressed.

***b. Isolation, pressure and their combined impact on mental health*.** Another barrier to gender equity identified by the PowerPack was the pressure faced by Black women scientists to excel and to achieve success and recognition in the face of structural and cultural barriers ( Figure 3D). PowerPack members reflected that this dynamic was strongly impacted by the intersection of race and gender. During colonization, Black Africans were not allowed to pursue scientific careers and only in recent generations have scientists of African origin been accepted into scientific institutions. PowerPack members noted that historical roots of non-acceptance subconsciously affect some African scientists and induce imposter syndrome that is independent of but compounds feelings brought on by gender discrimination. The uncomfortable and isolating experience of being an “only” (e.g. the only Black woman in a laboratory or a speaking panel) compounds this pressure and makes it more difficult to relieve some of these feelings through regular discussion with peers facing shared pressures. These dynamics and intersecting racial and gender biases exact pressure on African women scientists to work harder in order to achieve status that is achieved by members of other race and gender groups at a lower emotional cost. PowerPack members acknowledged that feelings of pressure were universal in science but agreed that pressure is felt unequally and especially affects Black African women scientists. Being a member of an identity group with limited traditional or current power may encourage scientists to try to deal with this pressure without involving others, “making trouble” or drawing attention to themselves. Scientists who have additional intersecting minoritized identities (e.g. sexual-orientation, gender or disability) may experience further isolation without having access to peers or role models who share similar experiences. Importantly, these pressures were noted to eventually interfere with mental health of Black women scientists and to have the potential to negatively affect their performance as scientists ( Figure 3D). 

***c. The perceived conflict between motherhood and science*.** Another barrier to gender equity is the idea that there is a conflict between being a scientist and being a mother, which can lead to female scientists leaving the field after becoming mothers ( Figure 3E–F). This barrier was noted to have effects at individual, environmental and institutional levels. Women PowerPack members reported often feeling pressure to conform to expectations to be constantly available for scientific work, to work extended hours, to travel, to compete for prestigious grants and faculty positions in order to maintain their professional identity, all while being fully present mothers. PowerPack members who had children or who wished to have children reported feeling that these twin ideals were unsustainable. PowerPack mothers reported that they struggled to request help from external sources for their childcare duties because asking for help prompted feelings of inadequacy within societies that exert unrealistic expectations upon women. Though the inability to cope with the combined pressures of motherhood and patriarchal institutional cultures can at times be perceived as an internal problem of the female scientist, external solutions are necessary. Lack of visibility of scientist mothers was noted to be an environmental barrier in scientific arenas and in the wider culture. At the institutional level, PowerPack members highlighted the lack of structural support for scientist-mothers at their institutions, and attributed lack of material support for mothers as one of the contributing factors to excellent female scientists exiting the field ( Figure 3F&G). Specific impediments to gender equity in science included absence of clear maternity leave policies for scientific trainees and early career researchers and lack of facilities for lactation upon return to work. Labor laws that accommodate parenthood have been enacted in most countries around the world ^22, 23^. However, PowerPack members reported that most of their scientific institutions are not structured to execute these policies.

### Solutions for gender equity in science

After identifying these barriers to gender equity in science the PowerPack proposed possible solutions. Much has been written about policy-level solutions and a comprehensive review is beyond the scope of this project ^24– 26^. Here we highlight particular solutions for pursuit of gender equity in African science that arose from the unique perspective of the PowerPack. Solutions included those that can be addressed by individuals, society and institutions.

***a. Actions women can take at the individual and collective level*.** Individuals have an important role in addressing gender inequity in science. Representation is one of most effective bridges to fight gender disparities, and can be achieved through peer support, mentorship, and public campaigns ( Figure 4A). Women scientists need to overcome fear and negative internal scripts that contribute to feelings of inadequacy and imposter syndrome. This negative ‘voice’ is often the result of previous ill-treatment, past trauma, internalized societal pressures and culture (both scientific and non-scientific). Senior PowerPack members made it clear that it is critical for women to find and use their voices in the scientific sphere. They emphasized the need for women to recognize the value of their perspectives to the scientific community and to become comfortable taking up physical and theoretical space. Claiming a seat at the table and giving voice to their perspectives will create a virtuous cycle that will change science and its gender norms ( Figure 4B–C). The positive experience of the internal discussions among PowerPack members during the African Scientists for Gender Equity campaign highlighted the power of single- and multi-generational peer support groups as a strategy to enhance gender equity in science. PowerPack members realized that gendered biases internalized during childhood will not disappear overnight and will require affected individuals to be intentional in overcoming and unlearning these through regular conversations in a supportive environment ( Figure 4A). Such discussion groups will also provide accountability and positive reinforcement of new thinking patterns. Multiple platforms have been established for African women, to encourage empowerment and accelerate women placement as directors and examples of those are Boardroom Africa, Gender Summit Africa etc. ^27^. Individual or groups sustainable mentorship programs through workshops and receiving professional guidance has been reported to improve women’s scientific outputs and should continue to be used as a tool to facilitate gender equity in science ^27, 28^.

It is also critical for women to completely re-write their own narratives around motherhood and science. Not only is it possible to combine the two, but it is also wonderful to do so! Without “sugar-coating” the inherent difficulties in pursuing two time-intensive vocations simultaneously, SuperScientists who are also mothers highlighted these important lessons: 1) understand the truth of the proverb "it takes a village to raise a child" and learn to solicit and accept support from family, friends, colleagues, and childcare providers; 2) accept that your journey as a mother and scientist is never going to be perfect so expect mistakes and avoid negativity and shame when they occur in either realm, ( Figure 4D); 3) encourage your children to understand the importance of your work so they will understand the reason why you are busy and respect your contributions to society; ( Figure 4E) 4) re-frame the pleasures of motherhood experienced on evenings and weekends as the "reward" for the hard work and long hours put into science, and vice versa. Overall, in trying to take these suggestions into action, discomfort will be experienced but one should remember that we have to be uncomfortable to make progress ( Figure 4F).

***b. The role of men in improving gender equity in science*.** The campaign identified environmental and institutional barriers to gender equity, which require advocacy and external engagement to address. For instance, while women must re-write the narrative of conflict between motherhood and science themselves, it is also critical for male scientists and the wider scientific culture to understand and promote the idea that women can pursue motherhood and science simultaneously and be successful in both realms. Male PowerPack members emphasized that gender equity in science was not just a problem for women but a concern for the entire scientific community. Due to the current underrepresentation of women in leadership positions, men currently hold the vast majority of power in African science and therefore must overcome feelings of awkwardness or defensiveness when addressing the issue and commit themselves and their institutions to improving gender equity in science. Mentors (male and females) need to intentionally provide opportunities to female mentees and promote their progression into leadership positions by embracing "sponsorship" as an alternative to traditional mentorship ^29^. Given that science in Africa is largely funded by donors, their increased attention to intersectional gender and other inequities within African science, could result in significant shifts. We suggest that funders examine the insights raised in this manuscript and designate substantial funds to the advancement of women scientists at all career levels. Funders need to be intentional about providing more funding opportunities to women scientist and using their funds to redress inequity in straightforward and creative ways. Male leaders should intentionally promote female successors for their positions and ensure that opportunities are offered to women and not informally arranged through traditional male-dominated networks ("Boys Clubs") ( Figure 5A). Male scientists must also reflect on their actions outside of the scientific environment and actively help to subvert their culture’s gendered stereotypes ( Figure 5B–D). Taking an active role in parenting, especially by taking paternity leave in the early newborn period, can be a powerful antidote to cultural beliefs about gendered roles in the domestic sphere ^23, 30^.

***c. The role of institutions in improving gender equity in science*.** Institutions must be intentional about addressing gender inequity in science and produce comprehensive rubrics to improve salary and funding parity, female representation in leadership, and gender equity policies within their organizations ( Figure 6A–B). Our campaign highlighted institutional solutions most relevant to Black women in the early stages of their scientific careers. The most critical policy improvements addressed the problematic idea of a conflict between motherhood and a successful scientific career. A very important recommendation was the need for scientific institutions to provide clear maternity leave policies, especially for trainees/post graduate students. There was also a need for providing child-care assistance for scientists traveling with their babies for scientific conferences or funds to cover extra childcare when children are left at home. In addition, the need for sanitary and comfortable lactation rooms in workplaces was highlighted. This is legislated in the labor laws of some countries, for instance the South African employment act legislates for breastfeeding/milk expression breaks for lactating mothers ^31^. However, a major challenge is that many employers and employees are not aware of these policies or their rights ^32^. In addition, although these suggested policies are very good, the lack of proper implementation, monitoring and tracking prevent success. Scientific institutions must implement progressive maternity and lactation policies and track their impact over time in order to retain women in the scientific career pipeline. Additionally, it is crucial for policy makers and institutions to (i) recognize intersectionality and its impact on power dynamics in scientific environments, (ii) be critical in examining internal biases and prejudices when leading institutions, and (iii) design and enact explicit policies to encourage and provide opportunities to people whose sex and race disadvantages them. Lastly institutions must provide opportunities to people according to merit and scientific capabilities and not according to their sex, pregnancy or motherhood status ( Figure 6C–D).

## Concluding remarks

This campaign harnessed creative and fun imagery to transform 24 African scientists into a PowerPack advocating for gender equity in science. By amplifying the voices of diverse scientists, particularly Black African female graduate students, who are often silenced by the culture of science, our campaign highlighted specific practices and policies that can be adopted to advance gender equity in science. The campaign highlighted the importance of including diverse groups of people in order to capture various perspectives when addressing equity issues. The PowerPack included scientists who were male and female, from diverse career stages, and from 6 sub-Saharan African countries. This diversity brought innovative ideas to our themed discussions and when combined with the superhero imagery resulted in a positive and empowered approach to topics which can often be heavy and difficult to discuss. This approach resulted in a number of insights that are immediately actionable by PowerPack members and their institutions. The campaign fostered a collaborative culture across race, gender and age lines and the PowerPack felt that such solidarity is imperative for true and lasting change to be implemented. The diversity of the group also allowed the PowerPack to appreciate the importance of conceptualizing solutions to gender equity that act at multiple levels. By listening attentively to the concerns articulated by the youngest and least systemically empowered members of the PowerPack, the group could strategically imagine those solutions that could be spearheaded by individual female scientists and those that would require advocacy by male allies to change scientific institutions. Identifying solutions at multiple levels gives a clear picture of what each stakeholder can contribute to advancing gender equity in science. This collaborative spirit, if adopted widely, has the potential to relieve female scientists of the pressure of advocating to pursue their careers against stark odds while also struggling to combat external factors that contribute to gender inequity in science.

This project formed a baseline for identifying and raising awareness on very important themes to promote gender equity in African science. We recognize that there are other barriers and challenges faced by women scientists and other marginalized groups, which may not necessarily be discussed in this work since we were unable to capture all geographic areas and cultures in Africa. Because this study was centered on the SANTHE network, its failure to include scientists from West and North African institutions, Francophone institutions and less well funded institutions limits the generalizability of the insights reported. More work needs to be done to develop viable interventions, while also identifying ways to implement all the recommended solutions identified from this campaign. Future work is also needed to investigate the effectiveness of interventions and how they promote women participation in science, technology, engineering and mathematics (STEM) and retention among women and girls.

**Figure 7.**
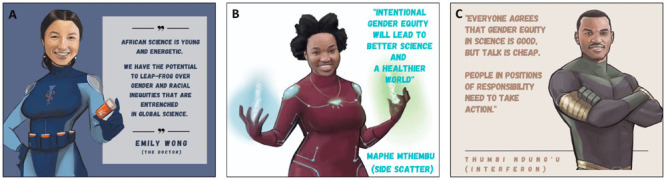
Key themes of the African Scientists for Gender Equity campaign.

African science is young and energetic and will substantially benefit from addressing the ills caused by gender inequity. Africa has the potential to leap-frog the rest of the world in its pursuit of a healthier, more diverse and innovative culture of science ( Figure 7A&B). Now is the time to act to address the barriers and enact the solutions highlighted in this innovative campaign. We challenge every stakeholder to take substantive action in the pursuit of gender equity in African science ( Figure 7C), as this is a matter of urgency to improve the quality and innovation of African science.

## Data Availability

No data are associated with this article.
