# Supplementary material for: A PowerPack of SuperScientists: An innovative concept by African scientists to address gender bias and inequity in science
Source: Wellcome Open Res. 2023 Feb 24;7:87. Originally published 2022 Mar 11. [Version 2] doi: 10.12688/wellcomeopenres.17668.2 (PMC10338983; doi:10.12688/wellcomeopenres.17668.2)
Supplement: Peer review history

# Peer review: Reviewer response for version 2

**DOI:** 10.21956/wellcomeopenres.20687.r59877

**Published:** 2023-07-12

**Authors:** Eirini Christou

**Affiliations:** 1 Cyprus University of Technology, Limassol, Cyprus

Overall, this work highlights an approach to addressing the under-representation of women in scientific leadership in sub-Saharan Africa. By involving diverse voices, particularly those typically marginalized in discussions of gender equity, and utilizing creative methods such as superhero imagery, the campaign aims to raise awareness, challenge stereotypes, and propose  solutions.

 Comments:

- The paper is well-written and easy to follow.
- How and why was this approach selected? Is there a rationale behind this? 
- The contribution and the aim of this work needs to be highlighted. How does this practice contribute to gender equity?
- Is there further info on the profile of the participants? e.g. scientific field?
- Table 1 -  For the suggested solutions, indicate whether they are extracted by the discussion or by literature (or both) and provide references (if applicable). 
- While it is mentioned that the campaign garnered a significant number of followers and impressions, it does not provide any details about the actual impact, engagement, or reach of the campaign.
- How were the superheroes created? What tools were used e.g. each scientist used a digital tool to create their own superhero? Did scientists work individually in creating their superhero or in groups? Synchronously or asynchronously?
- What are examples of questions(?) used to guide the closed sessions?
- How were the campaign themes extracted/analysed from the discussion? Was a specific method followed?

Does the article adequately reference differing views and opinions?

Partly

Are all factual statements correct, and are statements and arguments made adequately supported by citations?

Partly

Is the Open Letter written in accessible language?

Yes

Where applicable, are recommendations and next steps explained clearly for others to follow?

Yes

Is the rationale for the Open Letter provided in sufficient detail?

Partly

Reviewer Expertise:

gender equality,  research methodology, education

I confirm that I have read this submission and believe that I have an appropriate level of expertise to confirm that it is of an acceptable scientific standard, however I have significant reservations, as outlined above.

# Peer review: Reviewer response for version 2

**DOI:** 10.21956/wellcomeopenres.20687.r55208

**Published:** 2023-03-24

**Authors:** Millicent Liani

**Affiliations:** 1 International Institute of Tropical Agriculture, Oyo State, Nigeria

Many thanks for the opportunity to review this article. I hereby acknowledge that the authors have addressed all the comments based on my initial review. I, therefore, look forward to seeing this article indexed.

Does the article adequately reference differing views and opinions?

Partly

Are all factual statements correct, and are statements and arguments made adequately supported by citations?

Partly

Is the Open Letter written in accessible language?

Yes

Where applicable, are recommendations and next steps explained clearly for others to follow?

Yes

Is the rationale for the Open Letter provided in sufficient detail?

Yes

Reviewer Expertise:

Gender equitable scientific career pathways in Africa; research capacity strengthening; gender integration in research and development projects; intersectionality; gender-responsive and transformative research

I confirm that I have read this submission and believe that I have an appropriate level of expertise to confirm that it is of an acceptable scientific standard.

# Peer review: Reviewer response for version 1

**DOI:** 10.21956/wellcomeopenres.19545.r49169

**Published:** 2022-05-05

**Authors:** Millicent Liani

**Affiliations:** 1 International Institute of Tropical Agriculture, Oyo State, Nigeria

Many thanks for the opportunity to review this article. It was a pleasurable experience reading it! Great efforts by the authors to unraveling practical advice and actions towards helping women seeking to advance their scientific careers in Africa, a research area that has been largely anecdotally explored. I have minor comments and suggestions that should be addressed to make the article scientifically sound:

1. Throughout the paper, the authors use the term ‘fighting’ for gender equity in science, which from the discourse of gender and development, this term sounds derogative. Instead, they could use a better term and tone such as ‘advocating’ for gender equity in science.
2. The term ‘individual’ interventions should be used consistently throughout the paper as opposed to using it interchangeably with ‘internally’ as it is appearing in the abstract
  .
3. Re: the approach – Need to add that ‘Using an intersectionality lens’ (as this is clearly spelled out as a keyword in the abstract), we selected a group that represented diverse career stages…. (see my edits on pg. 3). The countries outlined are all Sub-Saharan Africa (SSA) so it could be important if the authors explicitly state that the 24 scientists represented six SSA countries. Additional information on how the study participants were selected, as well as their distribution across various career stages by gender, would be useful to illuminate variations amongst the participants.
4. The opening statement on the theme about ‘barriers to gender equity in science’ (pg 4) is hanging as it is unclear whether this is in SSA or globally. The authors could also consider providing some further nuanced reflections on how the sub-themes on ‘isolation, pressure and their combined impact on mental health’; ‘The perceived conflict between motherhood and science’; and ‘actions women can take at individual and collective level’ relates to existing SSA literature. This would greatly strengthen the claims being made in the paper.
5. Going through the references, about 75% of the cited literature is from the global North yet the paper is amplifying the experiences of researchers in the SSA region. The author/s could strengthen the results & discussion section to include insights from SSA studies.
6. Need to proofread the paper for typos and grammatical errors.

I look forward to seeing this important piece of work published!

Does the article adequately reference differing views and opinions?

Partly

Are all factual statements correct, and are statements and arguments made adequately supported by citations?

Partly

Is the Open Letter written in accessible language?

Yes

Where applicable, are recommendations and next steps explained clearly for others to follow?

Yes

Is the rationale for the Open Letter provided in sufficient detail?

Yes

Reviewer Expertise:

Gender equitable scientific career pathways in Africa; research capacity strengthening; gender integration in research and development projects; intersectionality; gender-responsive and transformative research

I confirm that I have read this submission and believe that I have an appropriate level of expertise to confirm that it is of an acceptable scientific standard, however I have significant reservations, as outlined above.

# Peer review: Reviewer response for version 1

**DOI:** 10.21956/wellcomeopenres.19545.r49172

**Published:** 2022-04-01

**Authors:** Faith Osier

**Affiliations:** 1 Faculty of Natural Sciences, Imperial College London, London, UK; 2 KEMRI-Wellcome Trust Research Programme, London, UK

This is a nicely written article that raises awareness on the issue of underrepresentation of women in scientific leadership from an African perspective. It describes a public engagement campaign led by early-career black female scientists fighting for gender equity in science. The PowerPack of SuperScientists utilizes superhero imagery to counter the imposter syndrome that is faced by many scientists, and particularly black females.  It also served to subvert traditional power structures based on age, race and sex.  Three themes were debated on social media i) women cultural stereotyping that limits women’s careers ii) the perceived conflict between family and career for and iii) solutions that could be adapted by key stakeholders.  The authors acknowledge the importance of engaging men to achieve wider societal change.

 Creating awareness on the issues of gender and inclusivity in science, and particularly in scientific leadership is important and strongly appreciated. The comments raised below are efforts to encourage the authors and readers to broaden the diversity of dialogue across the richness of the African continent.

**Geographical representation and religious orientation**

 The authors focused on members of the SANTHE network.  This is a highly selected and privileged group and one questions how representative this is of science in Africa.  While there was an effort to include people at all career stages, there are glaring omissions in terms of geographical representation – notably West and North Africa.  There is also a lack of representation of cultural and religious differences. For young girls growing up in Muslim countries where the tradition of covering up is the norm, seeing superhero Muslim women scientists would have a major impact.  Similarly, unpacking cultural stereotypes across the breadth of the continent will be equally powerful and can only meaningfully be captured by the “wearer of the shoe”.  Further collaboration across geographies in such initiatives would highly enrich debate and go further towards breaking down these stereotypes.

**Francophone and Lusophone Africa…and others**

 Related to geography is language.  Unfortunately, the experience of young girls aspiring to scientific leadership is to a large extent dictated by where they find themselves – and these colonial legacies continue to limit ambitions and opportunities. Career trajectories can be vastly different in the different settings and we need to be cognizant of this and work to address it. Funding opportunities are still dictated by colonial history and Africa needs to chart its own future in this regard and give equal opportunities to all its people.

**Funded-researchers privilege**

 While we often discuss the “older white men” privilege in science, we are in danger of overlooking our own privilege as black African women, working in highly networked and well-funded research groups. Is this representative of science in Africa?  For many striving female scientists across the continent, the main issue may be access to the basics, resources to undertake basic experiments, exposure to research infrastructure and mentorship that gives them a chance of realizing their ambitions. The societal system that created a lack of women in scientific leadership, is the same one that prevents many would-be scientists from seeing the light of day.  The inequity of education, physical security and opportunities for women vary greatly across the continent and those of us in the countries that participated in this article often take this granted. Listening to the other voices will add richly to the debate in ways that we cannot even consider.

**Superhero or teamwork?**

 Similarly, whilst I love and understand the desire to portray scientists as superhero’s – the concept of superhero’s generally speaks to particularly outgoing and highly visible personalities that singlehandedly “save the world”. This is more the realm of fiction than fact.  Science is a collaborative endeavor that requires teamwork across diverse personalities. The role of the technician who performs a routine task every single day should not be considered less than that of the team leader that gets awarded the prize. Portraying images of teams and people working together is perhaps something that the authors could consider in future iterations. The issue of personalities is important in science.  Like in many other areas, charismatic individuals that may not necessarily be the best scientists win the day and this perpetuates a lack of inclusion of different kinds of personalities that are equally vital to the scientific endeavor. How do we portray images of “strong silence” that nevertheless get the job done? How do we reach out to the different personalities that realistically reflect who we are?

**Preaching to the converted and measuring impact**

 How do we bring about societal change?  Who is responsible for bringing about change?  Who decides who gets to sit at the leadership table?  How does one engage institutional leaders? Are we talking just to ourselves? I commend the authors notes on actions that we can take as individuals – each one doing their part.  Those in leadership positions must work to bring up more women and to support them to remain. There is power in numbers.  Whilst a fair amount was discussed about what institutions should do, little was said on how this could happen, who would hold institutions accountable and will force be required? Is this a top priority for institutions in Africa? What needs to happen to make it a top priority?  Debate and papers such as this are a good way to start, but I felt more is needed to translate talk to action. 

**The role of funders**

 The authors should comment on the role that funders and funding organizations can play in promoting gender diversity and inclusivity. Given that science in Africa is largely funded by donors, they can insist on how the work they support is conducted.

**Gender diversity and inclusivity: urgent?**

 The authors should also take a stand on whether there is any sense of urgency to the challenge of underrepresentation of women in scientific leadership. Is this “a good thing to do” or is there an urgency?  Is this a good story to tell or is this change that we really believe is important, urgent and will have a major impact.

Does the article adequately reference differing views and opinions?

Partly

Are all factual statements correct, and are statements and arguments made adequately supported by citations?

Yes

Is the Open Letter written in accessible language?

Partly

Where applicable, are recommendations and next steps explained clearly for others to follow?

Partly

Is the rationale for the Open Letter provided in sufficient detail?

Yes

Reviewer Expertise:

Parasitology, Immunology, Vaccines

I confirm that I have read this submission and believe that I have an appropriate level of expertise to confirm that it is of an acceptable scientific standard, however I have significant reservations, as outlined above.
